# Vasculogenic Mimicry: Become an Endothelial Cell “But Not So Much”

**DOI:** 10.3389/fonc.2019.00803

**Published:** 2019-08-22

**Authors:** Mónica Fernández-Cortés, Daniel Delgado-Bellido, F. Javier Oliver

**Affiliations:** CSIC, CIBERONC, Instituto de Parasitología y Biomedicina López Neyra, Granada, Spain

**Keywords:** vasculogenic mimicry, tumor microenvironment, metastasis, VE-cadherin, anti-angiogenesis therapeutic failure, cell plasticity

## Abstract

Blood vessels supply all body tissues with nutrients and oxygen, take away waste products and allow the arrival of immune cells and other cells (pericytes, smooth muscle cells) that form part of these vessels around the principal endothelial cells. Vasculogenic mimicry (VM) is a tumor blood supply system that takes place independently of angiogenesis or endothelial cells, and is associated with poor survival in cancer patients. Aberrant expression of VE-cadherin has been strongly associated with VM. Even more, VE-cadherin has constitutively high phosphorylation levels on the residue of Y658 in human malignant melanoma cells. In this review we focus on non-endothelial VE-cadherin and its post-translational modifications as a crucial component in the development of tumor VM, highlighting the signaling pathways that lead to their pseudo-endothelial and stem-like phenotype and the role of tumor microenvironment. We discuss the importance of the tumor microenvironment in VM acquisition, and describe the most recent therapeutic targets that have been proposed for the repression of VM.

## Background

The concept of neovascularization was described for the first time in 1787 in the context of developmental biology. The term angiogenesis was coined early in the 20th century but was not applied to tumor biology until decades later (1). Angiogenesis can arise in a variety of forms during cancer, namely sprouting angiogenesis, intussusceptive microvascular growth, and glomeruloid microvascular proliferation (2). Emerging studies have shown that a few tumors can grow without need of angiogenesis even in hypoxic conditions, while other tumors display both angiogenic and non-angiogenic regions (3). Vasculogenic mimicry refers to the ability of cancer cells to organize themselves into vascular-like structures for the obtention of nutrients and oxygen independently of normal blood vessels or angiogenesis.

Research in VM has often been surrounded by skepticism to a certain extent. The reason for this controversy is usually the difficulty to distinguish VM channels from endothelial blood vessels or blood lakes *in vivo*. Similarly, *in vitro* research presents inherent inconveniences, given the need to develop 3-D models where tumor cells can develop capillary-like structures. Most *in vitro* research is based on cell cultures using matrigel. However, the tubular structures observed in this model might not always represent *in vivo* VM. Table 1 shows a few articles where VM is thoroughly described in various cancer types *in vivo* and *in vitro* (4–10, 12, 13).

**Table 1:** Main tumor types where VM has been reported and main inhibitors described against VM.

**Cancer type**	***In vitro* (Molecular target involved)**	***In vivo* (Molecular target involved)**
**Tumor types presenting VM**		
Breast	Liu et al. (4) (S1PR1/VE-cadherin)	Liu et al. (5) (CD133,MMP-2/9), Liu et al. (4) (S1PR1/VE-cadherin)
Colon	Qi et al. (6) (Wnt 3a, β-catenin, VEGFR1/2, VE-cadherin)	Qi et al. (6) (Wnt 3a, β-catenin, VEGFR1/2, VE-cadherin)
Glioblastoma multiforme		El Hallani et al. (7) (EphA2, laminin 5γ2, TFPI-1, Neuropilin-2, endoglin)
Hepatocellular carcinoma		Sun et al. (8) (Twist-1, VE-cadherin, MMP-9)
Lung cancer		Xia et al. (9) (Sema4D, MMP-2/9, VE-cadherin, EphA2, PlexinB1)
Melanoma		Dunleavey et al. (10) (CD31, tyrosinase, AP-2α)
Pancreas	Yang et al. (11) (HIF-2α, VE-cadherin, Twist1)	Yang et al. (11) (HIF-2α, VE-cadherin, Twist1)
Ovarian	Racordon et al. (12)	Tang et al. (13) (Urokinase, MMP-2, AKT)
Small cell lung	Williamson et al. (14) (VE-cadherin)	Williamson et al. (14) (VE-cadherin)
Ewing sarcoma	van der Schaft et al. (15) (TFPI-1, VE-cadherin, Integrin α3, EphA2)	van der Schaft et al. (15) (TFPI-1, VE-cadherin, Integrin α3, EphA2)
**Novel therapeutic agents**	**Molecular target or function**	**Effect on VM**
**Targeting VM**		
CMV-1118	Phospho-MAPK/phospho-kinases, cell stress checkpoints, and apoptosis regulators	Inhibition *in vitro*, phase I clinical trials with sorafenib (NCT03582618)
PF-562271	FAK	Inhibition *in vitro*
AKB-9978	VE-PTP/TIE-2	No data
STI-571	PDGF-β	Inhibition *in vitro* and *in vivo*

The unique arrangement of VM grids simulates embryonic vasculogenesis, suggesting that malignant tumor cells acquire an embryonic-like phenotype. Gene expression analysis showed that aggressive tumor cells capable of VM display a diversified gene profile, expressing genes from multiple cell types such as epithelial cells, fibroblasts and endothelial cells (16, 17). Nevertheless, the molecular mechanisms that give rise to VM remain largely unknown.

Vascular endothelial cadherin (VE-cadherin), also known as cadherin 5 or CD144, is a cell-cell adhesion protein commonly expressed by endothelial cells. Phosphorylation of VE-cadherin at a number of residues can modulate endothelial junction stability and permeability in different contexts (18). VE-cadherin was believed to be specific for endothelial cells (ECs), though in the past few decades it has been found in a wide variety of tumors, where it crucially contributed to aggressiveness and VM. In fact, VE-cadherin was found in highly aggressive tumor cells but it was not expressed by their poorly aggressive counterparts. Moreover, VE-cadherin down-regulation leads to the inhibition of VM formation (19). In this mini-review, we focus on the new findings on the role of extravascular VE-cadherin and its different localizations in the acquisition of the VM phenotype. In addition, we discuss the influence of the tumor microenvironment, stromal cells and mural cells in the development of VM.

## VE-Cadherin in Non-Endothelial Context

**Figure 1:**
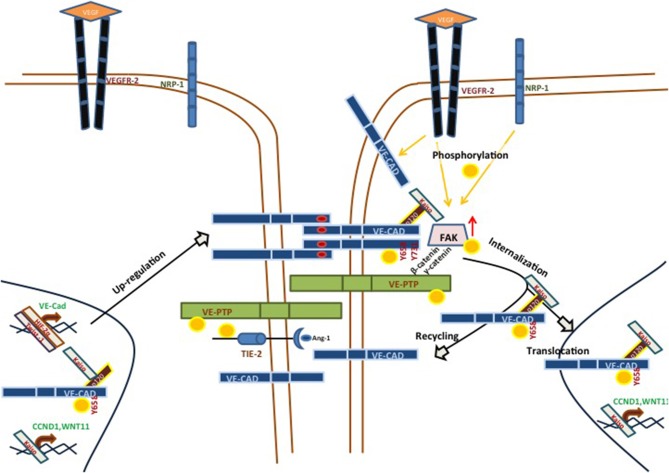
Main signaling pathways implicated in VE-cadherin/VM-positive cells. VE-cadherin can be phosphorylated by VEGFR-2, alone or in a NRP-1-dependent manner, in response to different VEGF soluble factors. These stimuli lead to the phosphorylation of VE-cadherin at Y658 and its subsequent internalization. pY658-VEC can interact with p120 and Kaiso. This complex prevents Kaiso from binding its target genes (CCND1, WNT11), promoting VM formation in aggressive melanoma models. VE-PTP may be an important factor in the maintenance of VE-cadherin phosphorylation status, and catenins p120 or β-catenin may influence the VE-PTP/VE-cadherin axis. On the other hand, VM-positive aggressive melanoma cells showed an up-regulation in a number of proteins, as compared with poorly melanoma cells. One of these proteins was TIE-1, which may be a target of VE-PTP, suggesting an implication in the TIE/angiopoietin pathway.

VE-cadherin is a single pass transmembrane protein typically found in endothelium, where it takes part in adherents junctions (20). VE-cadherin has been extensively studied with reference to vascular adhesion, but its function during VM in aggressive tumor cells is not fully understood. The structure of VE-cadherin contains five extracellular calcium-dependent domains (aminoacid residues 46-599) which can establish *cis* homodimers with another VE-cadherin molecule. Similarly, VE-cadherin can form *trans* dimers binding through the ECDI-ECDIV domains. The intracellular domain of VE-cadherin can undergo several post-translational modifications and 13 different residues of VE-cadherin have been reported to undergo phosphorylation in humans. Of these, residues Y658, S665, Y685, and Y731 have drawn most of the attention. They have been implicated in the intracellular dynamics of the cytoskeleton that control endothelial permeability. In particular, phosphorylation of VE-cadherin can trigger junctional changes via VE-cadherin internalization. As a result, vascular permeability is increased, allowing intravasation and extravasation of different cell types, including tumor cells (21). Recent investigations demonstrated that focal adhesion kinase (FAK) can phosphorylate VE-cadherin at Y658 in tumor-associated ECs, pinpointing the importance of FAK in regulating EC barrier function and hence tumor metastasis (22). FAK is a cytoplasmic tyrosine kinase co-activated by vascular endothelial growth factor receptor (VEGFR) 2 and integrin in the control of vascular permeability (23). Recently, we reported that human aggressive melanoma cells have a constitutively high FAK-dependent phosphorylation of VE-cadherin at Y658 (pY658-VEC). pY658-VEC interacts with p120-catenin and the transcriptional repressor kaiso in the nucleus. The inhibition of FAK led to the release of kaiso, promoting its recruitment to kaiso binding sites and therefore repressing kaiso target genes. Moreover, the repression of kaiso target genes CCDN1 and WNT11 abrogated VM. In that line, uveal melanoma cells genetically deficient for VE-cadherin (either through CRISPR/Cas9 technology or after silencing of VE-cadherin) lost the ability to develop VM. Even more, the rescue of WT-VE-cadherin reverted the ability to form VM; in contrast, expression of the non-phosphorylated Y658F-VE-cadherin blunted *in vitro* VM (24) (see Figure 1).

The intracellular domain V of VE-cadherin is necessary to bind vascular endothelial protein tyrosine phosphatase (VE-PTP) (25), in a plakoglobin (γ-catenin)-dependent way (26, 27). VE-PTP is an endothelial receptor-type phosphatase which was first described in relation to its implications in embryonic vasculature. VE-PTP-deficient mice undergo vasculogenesis but still die at the embryonic stage due to angiogenesis malfunction (28). VE-PTP may have many other implications, such as ocular vascular pathology (29), blood vessels development (30), breast cancer vasculature and metastatic progression (31). VE-PTP is also involved with the TIE1/2-Angiopoietin pathway. Different laboratories have shown that targeting VE-PTP with a specific inhibitor (AKB-9978, Aerpio Pharmaceuticals) activates TIE2 and stabilizes the ocular vasculature in ischemic/inflammation models (29). AKB-9778 induced TIE2 phosphorylation, directly as well as *via* ANG1. Furthermore, other signaling components of the TIE2 pathway, such as ERK, AKT, and eNOS (see Figure 1), also display increased phosphorylation (29). In 2015, Gong et al. reported that hypoxia increased the expression of VE-PTP in acute lung injury in a HIF-2α-dependent manner (32). All of these functions of VE-PTP have always been studied in endothelial models, though the implications of the VE-PTP/VE-cadherin axis in a VM context remain unknown. In view of the anomalously high levels of phospho-VE-cadherin in cells undergoing VM and the role of VE-PTP in the exquisite maintenance of VE-cadherin phosphorylation, new studies should address the function of this phosphatase in regulating vasculogenic mimicry.

## Tumor Microenvironment and VM

The complexity of tumors has been increasingly acknowledged in the past decades, to the point where numerous articles published in the cancer research field are no longer focused exclusively on cancer cells. On the contrary, the different components of the tumor microenvironment have received ever greater attention.

Low oxygen concentration in tumors, commonly known as tumor hypoxia, has been repeatedly associated with malignancy, metastasis and therapy resistance in cancer (33). Hypoxia has been linked to VM by many research groups as well (15). In hepatocellular carcinoma, hypoxia promoted VM through transcriptional co-activation of Bcl-2 and Twist1 (34). Nuclear co-expression of Bcl2 and Twist1 correlated with VE-cadherin expression in tumor cells. In fact, VE-cadherin gene expression can be induced by hypoxia, specifically by hypoxia inducible factor (HIF) 2α (35). Though it was first described in ECs, hypoxia-driven expression of VE-cadherin has been reported in a large number of tumor types too (36–38), where it is always involved in a promotion of the VM phenotype.

Hypoxia has been shown to promote VM through other signaling pathways apart from VE-cadherin. For instance, reactive oxygen species (ROS)-mediated stabilization of HIF1α activated the met proto-oncogene, which induced *in vitro* tube formation on matrigel in melanoma cells (39). Moreover, HIF1- and HIF2α promoted *in vitro* tube formation on matrigel through the upregulation of vascular endothelial growth factors (VEGF) C and D, as well as VEGF receptor (VEGFR)3 (40). In triple negative breast cancer, hypoxia increased the subpopulation of CD133^+^ cells (commonly regarded as cancer stem cells) through a Twist1-mediated mechanism. This population shift seemed to enhance tube formation, since CD133^+^ cells were found to line the VM-like tubes (41). In addition, HIF1α could promote tube formation in hepatocellular carcinoma by up-regulating lysyl oxidase like 2 (LOXL2) (42, 43), which is involved in collagen cross-linkage during extracellular matrix (ECM) remodeling.

ECM *per se* can play a fundamental role in regulating VM. The NC11 domain in collagen XVI could trigger tube formation in oral squamous cell carcinoma, since it could induce VEGFR1/2 expression (43). On the contrary, the presence of collagen I altered the vascular potential of pancreatic ductal adenocarcinoma (PDAC) CSCs, decreasing the secretion of pro-angiogenic factors and the expression of VEGFR2, altogether hindering VM formation in PDAC (44). Furthermore, Velez et al. showed that ECM architecture can influence VM; in particular, collagen matrices with small pores and short fibers induced β-integrin expression and hence VM (45).

The importance of non-cancer cells within the tumor stromal is slowly gaining attention in the study of VM as well. Tumor-associated macrophages (TAMs) seemed to promote VM formation in glioblastoma multiforme, namely increasing the expression of cyclooxygenase 2 in the tumor cells (46). Cancer-associated fibroblasts (CAFs) can be determinant in VM formation too. In a recent study, vasculogenic murine melanoma cells were injected in mice carrying a CAF-specific deletion for the matricellular protein CCN2. As a result, the absence of fibroblast-derived CCN2 reduced tumor vasculature, including VM (47). Finally, a recent publication by Thijssen et al. (48) showed that PAS^+^ tissues in human cutaneous melanoma stained positive for pericyte marker α-smooth muscle actin (αSMA) within the ECM networks lined by tumor cells. Furthermore, when VM^+^ tumor cells were co-cultured with pericytes, there was a stabilization of the VM networks for up to 96 h. Pericyte recruitment to VM networks was shown to be dependent on PDBF-B signaling, whereas the addition of STI-571 (imatinib mesylate) to inhibit PDGF receptor hindered VM as well as tumor growth.

## Targeting VM and Perspectives

A meta-analysis of 22 clinical studies derived from data concerning VM and 5-year survival of 3,062 patients across 15 cancer types showed that tumor VM is correlated with poor prognosis (49). Anti-angiogenic therapies (preferably, antibodies against VEGF receptor bevacizumab and related) against tumor development have had limited results so far. Therefore, the development of novel anti-tumor neovascularization strategies is of vital importance, expanding the targets from conventional angiogenesis to all the alternative mechanisms recently discovered, such as VM (50).

A unique small molecular compound with particular interest in anti-VM cancer treatment is CVM-1118, which is currently undergoing clinical trials (NCT03582618). CVM-1118 is classified as a phenyl-quinoline derivative, whose core structure displays potent anti-neoplastic and anti-mutagenic properties (51).

As mentioned above, pharmacological inhibition of activity of FAK/Y658 VE-cadherin with PF-271 may represent a new therapeutic opportunity in the repression of genes involved with VM promotion in cancer cells. Similarly, inhibition of the VE-PTP/TIE-2 pathway with AKB-9778 could open new ways to control the capacity to form pseudo-vessels by vascular mimicry cells. Finally, new treatments targeting mural cells, such as pericytes, could also have therapeutic value. It is the case of targeting PDGF-B axis with STI-571, which proved useful in VM mice models. Targeting VM with specific molecular compounds combined with front-line therapies may represent the best approach to obtain a good prognosis in patients in the future.

## Author Contributions

MF-C and DD-B designed and wrote the review. FO designed, wrote and coordinated the review.

### Conflict of Interest Statement

The authors declare that the research was conducted in the absence of any commercial or financial relationships that could be construed as a potential conflict of interest.
